# Effectiveness of Electrolyzed Water on Inactivation of Coliform Bacteria on Sweet Basil Consumed by Children and Estimation of Health Risk Assessment

**DOI:** 10.1002/mbo3.70112

**Published:** 2025-11-04

**Authors:** Gholamreza Khademi, Majid Sezavar, Maziar Naderi, Roghayeh Abedini

**Affiliations:** ^1^ Clinical Research Development Unit of Akbar Hospital Faculty of Medicine Mashhad University of Medical Sciences Mashhad Iran; ^2^ Department of Environmental Health Engineering School of Public Health, Tehran University of Medical Sciences Tehran Iran; ^3^ Division of Food Safety and Hygiene, Department of Environmental Health Engineering School of Public Health, Tehran University of Medical Sciences Tehran Iran

**Keywords:** children, coliform bacteria, fresh‐cut vegetables, human risk assessment, neutral electrolyzed water, sweet basil

## Abstract

This study focused on the effectiveness of neutral electrolyzed water (NEW) in inactivating coliforms on sweet basil, a fresh‐cut vegetable consumed, and estimating the human health risks related to the consumption of untreated sweet basil. The sweet basil samples contaminated with coliforms were treated with NEW to evaluate its effectiveness in inactivation. The hazard quotients (HQs) for untreated and treated sweet basil were then calculated based on the estimated daily intake and the toxicological reference values for coliforms. The results demonstrated that NEW was effective in reducing the coliform counts on sweet basil. The ranges of the total and fecal coliform removal efficiency were 62.31%–100% and 40.86%–100%, respectively. Moreover, HQ calculations indicated that consumption of untreated sweet basil had a higher risk of coliform exposure compared with treated sweet basil. On the basis of the results, NEW greatly reduced the risk of gastroenteritis in children (HQ decreased from 558,000 to 27.4). In other words, this disinfection solution reduced the probability of risk by 232,500 times. The application of NEW promises to reduce the microbial load on freshly chopped vegetables, such as sweet basil, making them safer for consumption, especially for children.

## Graphical abstract

Electrolyzed water effectively reduces coliform contamination on sweet basil, significantly decreasing health risks and potential gastroenteritis in children, thereby enhancing food safety for fresh produce consumption.

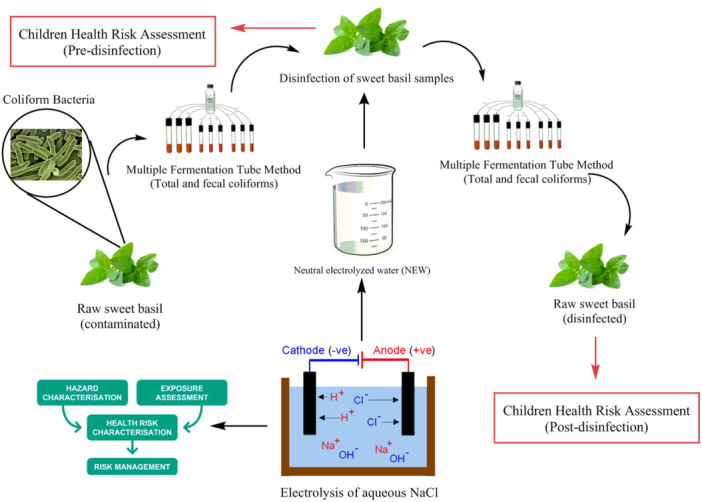

**Abbreviations**
ACMSF: Advisory Committee Microbiological Safety of Food
ADD: average daily dose
AEW: acidic electrolyzed water
ANOVA: analysis of variance
BEW: basic electrolyzed water
CCD: central composite design
CFU: colony forming unit
ED: experimental design
EW: electrolyzed water
HQ: hazard quotient
MPN: most probable number
NEW: neutral electrolyzed water
ORP: oxidation–reduction potential
RfD: reference dose
RSM: response surface method
TDI: tolerable daily intake

## 1 Introduction

Fresh herbs are widely used in culinary practices and are often consumed raw, which can pose significant risks of microbial contamination (Tresch et al. 2024). Among the pathogens of concern, coliform bacteria serve as key indicators of fecal contamination and overall hygiene quality of produce (Simiyari et al. 2024). The contamination of herbs with coliform bacteria can lead to foodborne illnesses, especially in vulnerable populations, such as children. Therefore, ensuring the microbiological safety of herbs is essential to protect public health (Florez et al. 2020; Mirahmadi et al. 2025; Hooshangi et al. 2024).

Fresh‐cut vegetables are a popular and healthy choice for children's meals. However, they can also harbor potentially harmful bacteria, including coliforms, which pose health risks if consumed in contaminated form (Osafo et al. 2022; Abedini et al. 2023). Similarly, fresh sweet basil (*Ocimum basilicum* L.) is a popular and nutritious addition to children's diets (Beuchat 2002; Abedini et al. 2021). When contaminated with coliforms, sweet basil can cause gastrointestinal infections, including diarrhea, vomiting, and dehydration (Bardsley et al. 2019). Children are particularly susceptible to diseases caused by contaminated fresh vegetables due to their immature immune systems (Berhanu and Pal 2020; Obande et al. 2023).

In recent years, neutral electrolyzed water (NEW) has emerged as a safe and effective method for disinfecting fresh produce (Naderi et al. 2024; Miranzadeh 2016). This type of water is created when the electrolysis cell operates without a membrane, resulting in a solution with a neutral pH of approximately 7 (Naderi and Nasseri 2020; Naderi et al. 2021). NEW is nontoxic and environmentally friendly, and its neutral pH ensures that it does not alter the organoleptic properties—such as color, aroma, taste, or texture—of the food (Soltani et al. 2024). Research has demonstrated that NEW can effectively eliminate a wide range of bacteria, viruses, and parasites from various food surfaces (Past et al. 2016; Saravani et al. 2020). The successful application of NEW in the food industry has made it a valuable disinfectant for reducing microbial contamination on fresh produce, including vegetables (Naderi et al. 2017, 2023).

Numerous studies have indicated that electrolyzed water is effective for a variety of fresh and fresh‐cut products, including carrots, radishes, lettuce, cucumbers, spinach, and broccoli (Naderi and Nasseri 2020; Iram et al. 2021). For instance, Plesoianu et al. found that after 14 days of refrigeration, fresh‐cut apples treated with NEW exhibited significantly greater firmness, total phenolic content, and antioxidant activity compared with those treated with other disinfectant solutions (Plesoianu 2021). Al‐Haq et al. (2005) demonstrated that during the electrolysis process, ozone (O_3_), hydrogen peroxide (H_2_O_2_), and hydroxyl radicals (OH) are generated, which contribute to the antimicrobial properties of electrolyzed water. Some researchers have suggested that bacterial inactivation primarily relies on oxidation–reduction potential (ORP) rather than residual chlorine levels. Additionally, Gómez‐López (2012) proposed specific operational parameters for using electrolyzed water to disinfect fresh‐cut vegetables contaminated with *Escherichia coli* O157:H7. In another investigation, Klintham et al. (2018) conducted a study on a two‐step washing process utilizing commercial vegetable washing solutions, along with electrolyzed oxidizing microbubble water, to decontaminate sweet basil and Thai mint.

One promising approach to reducing microbial loads on fresh produce is the application of electrolyzed water, a sanitizing agent known for its effectiveness, safety, and environmental friendliness (Miranzadeh 2016; Naderi et al. 2023; Gholami et al. 2019). Electrolyzed water contains reactive species capable of inactivating bacteria, including coliforms, thereby lowering the risk of foodborne disease transmission (Naderi 2017; Miranzadeh et al. 2021).

Health risk assessment methods are used to assess the safety consequences of consuming processed medicinal plants (Abedini et al. 2023; Gholami et al. 2018). A key component of this assessment is the hazard quotient (HQ), which quantifies the potential health risk associated with exposure to contaminants (Askari et al. 2024; Mohammed et al. 2025). The HQ is calculated as the ratio of the estimated exposure dose to a reference dose (RfD); an HQ value greater than 1 indicates a potential health concern, whereas a value below 1 suggests acceptable risk levels (Demissie et al. 2024; Farahmandkia et al. 2025).

The previous studies have demonstrated encouraging results regarding the ability to inactivate coliform bacteria and enhance the safety of fresh‐cut vegetables (Meghwar et al. 2024). While earlier research has confirmed the efficacy of NEW in reducing pathogenic microorganisms on various food surfaces, there remains a notable gap in the literature concerning its effectiveness specifically against coliform contamination in sweet basil (Nyamende et al. 2024). Few studies have systematically evaluated the application of NEW for managing coliform bacteria on herbs consumed by children, and conflicting findings exist regarding optimal treatment conditions and residual microbial reduction (Tango et al. 2019; Mugabe et al. 2024). Addressing this gap is crucial for establishing safe processing protocols for herbs like sweet basil in pediatric dietary contexts

This study aimed to assess the effectiveness of electrolyzed water in inactivating coliform bacteria on sweet basil consumed by children and to evaluate the associated health risks through the calculation of the HQ. By integrating microbial inactivation data with health risk assessment, this study provided a comprehensive evaluation of the safety and potential health implications of using electrolyzed water as a sanitizing treatment for fresh herbs.

## 2 Materials and Methods

### 2.1 Study Design

This study employed an experimental design to evaluate the efficacy of NEW in reducing coliform bacteria on sweet basil. The study also included a health risk assessment component to estimate the health risks related to the consumption of contaminated sweet basil.

### 2.2 Vegetable Samples Preparation

The fresh sweet basil was purchased from a local supermarket in Tehran city. The type of the prepared sweet basil in the study was a widely consumed sweet basil that is eaten raw with traditional foods, especially in Iran. The area of the sweet basil leaves was approximately 10 cm^2^ and the height was 10–30 cm. The sweet basil samples were stored at 10°C and used within 1 day. The vegetable samples without washing and reduction in size were stored in separate packages of 15 g in the refrigerator until disinfection by electrolyzed solutions.

### 2.3 Production of NEW

The new solutions were created using NaCl salt at concentrations of 0, 5, 10, 15, and 20 g/L, with a purity level of 99.5% sourced from Merck Company. An adjustable DC power supply was utilized to apply various electric potentials ranging from 0 to 30 V, with a current intensity of 0–5 A. The active chlorine concentration was subsequently assessed using a colorimetric method with a DR5000 spectrophotometer from Hach Company.

### 2.4 Disinfection of Sweet Basil Samples

**Figure 1:**
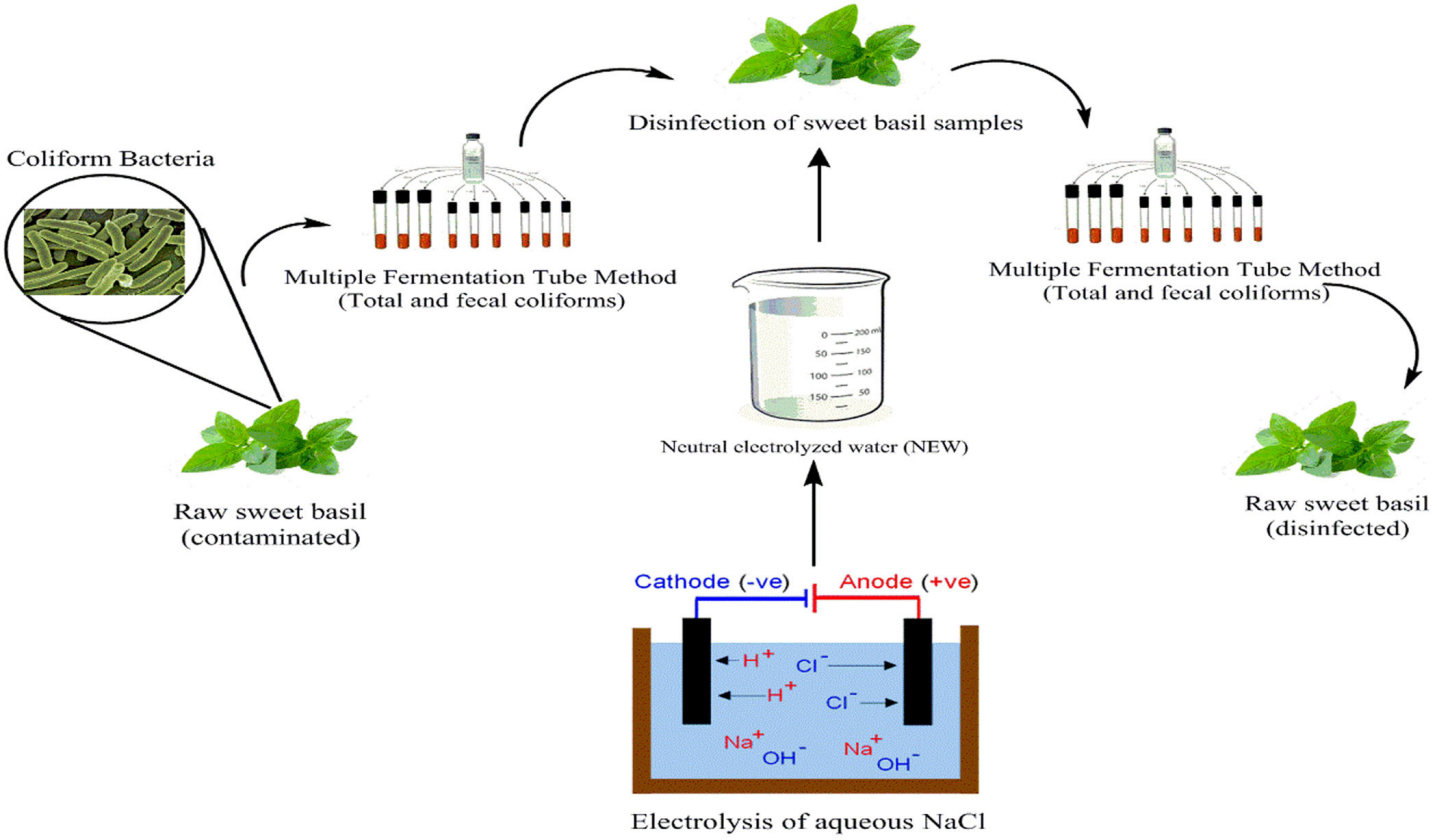
Stages of the study: the disinfection of fresh sweet basil by NEW and the evaluation of bacteriological contamination. NEW, neutral electrolyzed water.

To determine the initial contamination of fresh sweet basil as a control sample, 15 g of sweet basil was soaked in 900 mL of tap water. After 5 min of holding time, the water was tested for microbial contamination. The electrolyzed water was prepared at different NaCl concentrations (0, 5, 10, 15, and 20 g/L), at various electric potentials (3, 6, 9, 12, and 15 V), and during several electrolysis times (2.5, 5, 7.5, 10, and 12.5 min). For each test, 15 g of sweet basil were soaked in 900 mL of tap water. After each electrolysis step, 100 mL of the generated NEW was sampled from the cell by pipette and mixed with the sweet basil samples soaked in the above water. After 5 min of disinfection, 1 mL of water was sampled from each container and then transferred to the culture medium to count coliform bacteria (Figure 1).

### 2.5 Evaluation of Bacteriological Contamination

To enumerate the total and fecal coliform bacteria, we used the Multiple Fermentation Tube or Most Probable Number (MPN) method (Baird 2017). In the presumptive phase, the water samples were inoculated on lauryl tryptose broth medium by the multiple‐tube method. After incubating the tubes at a temperature of 35°C ± 0.5°C for 24–48 ± 2 h, the tubes containing gas and turbidity indicated the presence of total and fecal coliforms. In the confirmation phase, to confirm total and fecal coliforms, positive samples of the presumptive phase were inoculated in BGB and EC culture mediums, respectively. The samples added in the BGB broth were incubated at 35°C ± 0.5°C for 24–48 ± 2 h, and those added in the EC broth culture medium were incubated at 44.5°C ± 2°C for 24 h in Ben Marie. Eventually, the MPN number per 100 mL was obtained from the MPN table for total and fecal coliforms. The following formula was used to calculate the removal efficiency of total and fecal coliforms (Baird 2017):

$$\eta \;(\%)=\frac{\mathrm{Fecal}\mathrm{or}\;\mathrm{Total}\;\mathrm{Coliform}\;(\text{control})-\mathrm{Fecal}\mathrm{or}\;\mathrm{Total}\;\mathrm{Coliform}\;(\mathrm{sample})}{\mathrm{Fecal}\;\mathrm{or}\;\mathrm{Total}\;\mathrm{Coliform}\;(\text{control})}\times 100,$$

η (%) = the removal efficiency of total and fecal coliforms,

Fecal or Total Coliform (control) = the total and fecal coliform counts of the control sample,

Fecal or Total Coliform (sample) = the total and fecal coliform counts of the sample.

### 2.6 Experimental Design

The determination of the number of experiments and the statistical analysis of the data were carried out using experimental design and the response surface method (RSM). The number of trials was established using a central composite design, as shown in Table 1. Taking into account the minimum and maximum values for initial sodium chloride concentration (5 and 15 g/L), electric potential (6 and 12 V), and electrolysis duration (5 and 10 min), along with the central points, a total of 20 experiments were conducted.

**Table 1:** Input parameters and abbreviations for the exposure assessment of coliforms.

Parameters	Units	Values	References
Concentration of contaminant (*C*)	CFU/g	See Table 2	This study
Ingestion rate (IngR)	L/day	300 g/day	USEPA
Exposure frequency (EF)	Days/year	365	USEPA
Exposure duration (ED)	Years	6 years	USEPA
Body weight (BW)	kg	15 kg	USEPA
Averaging lifetime (AT)	Days	15 × 365	USEPA

### 2.7 Statistical Analysis

In this study, *Kolmogorov–Smirnov test* was employed to assess the normality of the data distribution. For the ED, statistical analysis, and optimization of removal efficiency for total and fecal coliforms, RSM was employed using *R version 3.6.2*. Additionally, *multiple‐way analysis of variance (ANOVA) test* was conducted to examine interactions between process variables. To assess differences in microbial contamination levels among samples, a multiway ANOVA was performed. For multiple comparisons, *Tukey's HSD test* was applied. Data analysis was carried out using *SPSS software version 21*, with *p* values less than 0.05 considered statistically significant.

### 2.8 Children Health Risk Assessment

The HQ was determined by dividing the estimated daily intake of coliform bacteria by the RfD set by regulatory bodies. For evaluating the risk and determine the probability associated with coliform bacteria in the sweet basil samples, the average daily potential dose (ADD) for both adults and infants was calculated for each brand using the following formula (EPA 2011):

(1) $$\text{ADD}\;=\frac{C\cdot \text{IngR·EF·ED}}{\text{BW·AT}},$$

where ADD represents the average daily potential dose in milligrams per kilogram per day (mg/kg‐day). The concentration of the contaminant is measured in milligrams per liter (mg/L), while the ingestion rate is quantified in liters per day (L/day). Exposure frequency is expressed as the number of days per year (days/year), and exposure duration is given in years. Body weight is measured in kilograms (kg), and the averaging time is recorded in days. The assumptions and their corresponding values used for calculating chronic daily intake via oral consumption are detailed in Table 1.

### 2.9 Hazard Quotient

The health risk associated with coliforms was evaluated using an HQ, which represents the ratio of coliform exposure to the tolerable daily intake (TDI) or RfD. For each sample, the HQ was determined by comparing the estimated ADD of coliforms ingested from contaminated sweet basil to the RfD for children (Bamuwamye et al. 2015; WHO 2009).

(2) $$\text{HQ}\;=\frac{\text{ADD}}{\text{RfD}},$$

where ADD and RfD (or TDI) are expressed in mg/kg‐day. In this study, the RfD of coliforms was considered according to the standards of the Food and Drug Administration (FDA) (EPA 2011).

## 3 Results

### 3.1 Disinfecting Power of the Produced Electrolyzed Water

The initial microbial load in the control sample (tap water with fresh sweet basil) was substantial, with total coliform counts averaging 1.3 × 10^6^ MPN/100 mL and fecal coliform counts averaging 9.3 × 10^4^ MPN/100 mL (Table 2). These baseline levels underscore the necessity for effective disinfection methods.

**Table 2:** The changes in the total and fecal coliform populations in the sweet basil samples.

Test No.	NaCl Co. (g/L)	Electric potential (V)	Electrolysis time (min)	Final total coliforms (MPN/100 mL) Initial: 1.3 × 10^6^ MPN/100 mL	Final fecal coliforms (MPN/100 mL) Initial: 9.3 × 10^4^ MPN/100 mL
1	10	9	7.5	540	130
2	15	12	10	140	10
3	15	12	5	350	4
4	10	9	7.5	540	130
5	5	12	10	230,000	18,000
6	5	6	5	400,000	55,000
7	5	6	10	490,000	20,000
8	5	12	5	330,000	54,000
9	15	6	10	130	130
10	10	9	7.5	130	130
11	15	6	5	1600	170
12	10	9	7.5	130	130
13	10	9	12.5	49	33
14	0	9	7.5	1,300,000	93,000
15	10	3	7.5	1,300,000	93,000
16	10	9	2.5	1600	350
17	10	9	7.5	1600	350
18	20	9	7.5	79	79
19	10	9	7.5	280	540
20	10	15	7.5	12	32

Abbreviations: Co., concentration; MPN, most probable number.

The produced electrolyzed water demonstrated significant microbial reduction under various optimized conditions. Notably, at an electrolyte concentration of 15 g/L, a voltage of 12 V, and an electrolysis duration of 10 min, the total coliform count decreased from 1.3 × 10^6^ MPN/100 mL to approximately 140 MPN/100 mL, representing over a 99.99% reduction. Similarly, fecal coliforms were completely inactivated, reducing from 9.3 × 10^4^ MPN/100 mL to zero within the same conditions.

In another condition, with 10 g/L NaCl, 15 V voltage, and 7.5 min of electrolysis, total coliforms decreased from 1.3 × 10^6^ to 12 MPN/100 mL, and fecal coliforms declined from 9.3 × 10^4^ to 32 MPN/100 mL, indicating substantial microbial inactivation. At 9 V, 12.5 min and 10 g/L NaCl, reductions to 49 MPN/100 mL (total coliforms) and 33 MPN/100 mL (fecal coliforms) were observed, further confirming the efficacy of the produced electrolyzed water.

**Figure 2:**
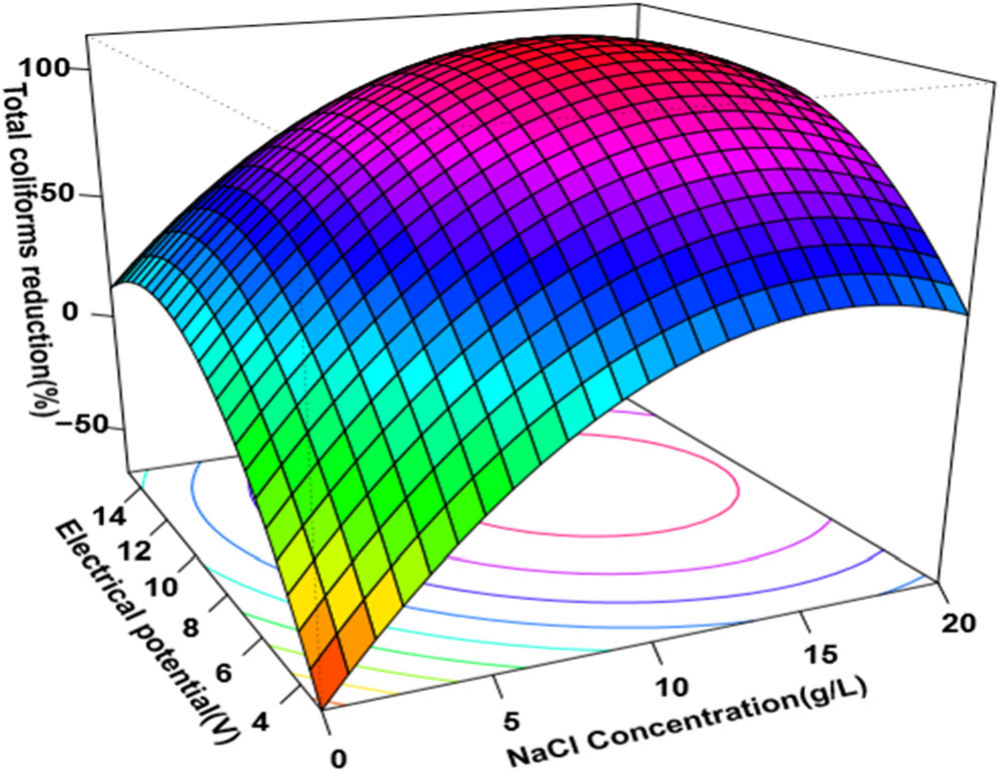
Response surface method of the total coliforms reduction as a function of electrical potential and the concentration of sodium chloride.

**Figure 3:**
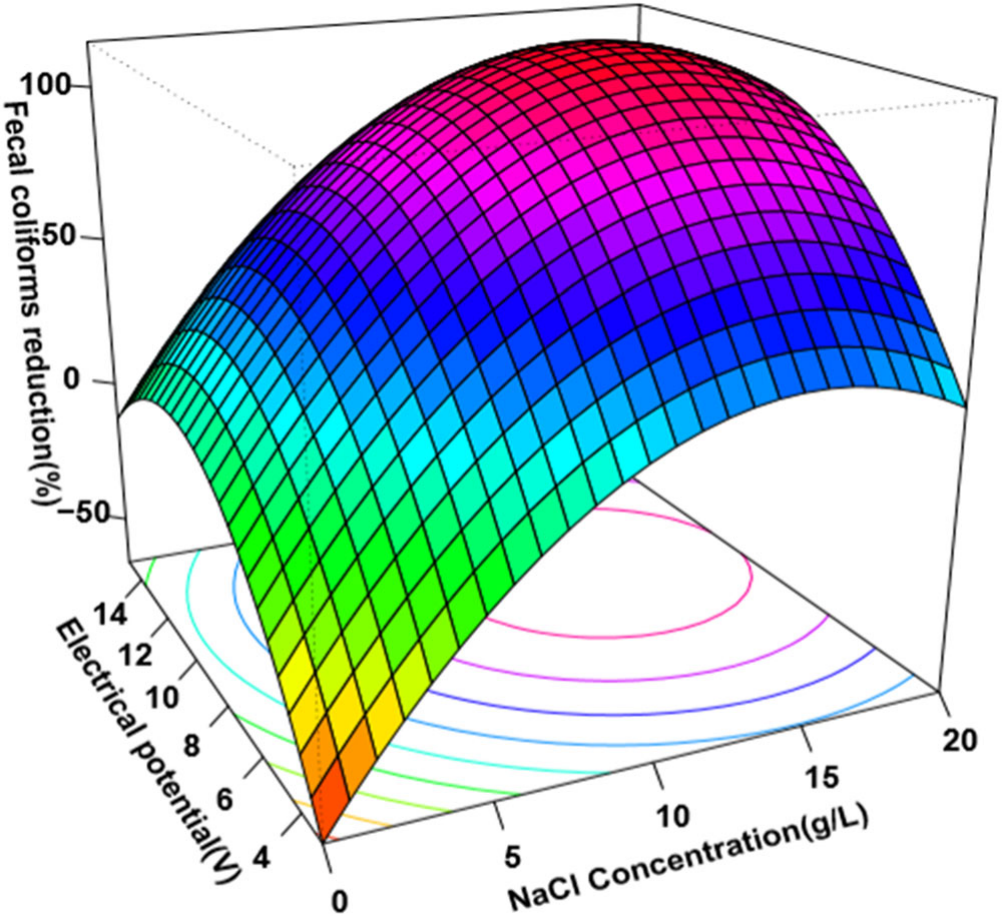
Response surface method of the fecal coliforms reduction as a function of electrical potential and the concentration of sodium chloride.

**Figure 4:**
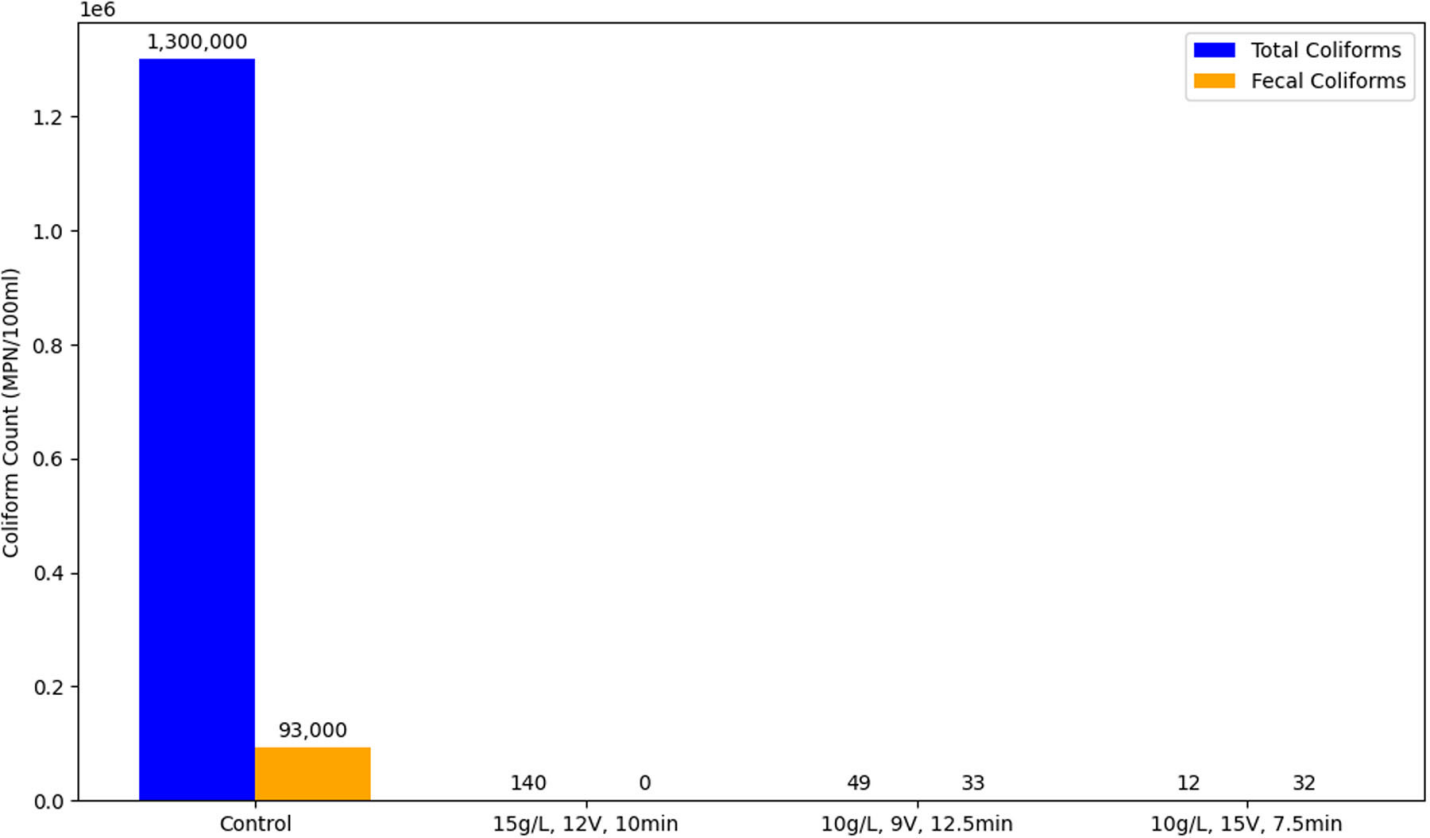
Effect of the electrolyzed water on coliform count.

Additionally, regression models depicted in Figures 2 and 3 illustrate a strong correlation between the reduction of coliform bacteria and variables, such as electrical potential and salt concentration. These models revealed that increasing voltage and electrolyte concentration enhances microbial inactivation efficiency. Moreover, the grouped bar chart in Figure 4 visually summarizes the significant reductions across various treatment conditions, emphasizing the optimized parameters for maximum disinfection efficacy.

### 3.2 Health Risk Assessment

Focusing on *E. coli* as the indicator pathogen, the initial contamination level in basil (control sample) was approximately 930 CFU/g (derived from 93,000 MPN/100 mL). On the basis of FDA standards, the permissible limit in fresh produce is 3 CFU/g.

Considering a 6‐year‐old child weighing 15 kg, the RfD for *E. coli* was calculated as follows:

$$\mathrm{RfD}=\frac{3\;\mathrm{CFU}/g}{15\;\;\mathrm{kg}},$$

RfD=0.2 CFU/kg.

Assuming daily consumption of 300 g of basil, the ADD before disinfection was

$$\text{ADD}\;=\frac{930\;\text{CFU}/g\times 300\;\;\text{g}/\text{day}\times 365\;\text{days}/\text{year}\times 6\;\text{years}}{15\;\mathrm{kg}\times 365\;\mathrm{days}\times 1000\;g/\mathrm{kg}}.$$

The HQ before disinfection was thus

$$\mathrm{HQ}=\frac{\mathrm{ADD}}{\mathrm{RfD}},$$

$$\mathrm{HQ}=\frac{111\text{,}600}{0.2},$$

HQ=558,000.

Postdisinfection, the *E. coli* level was reduced to approximately 4 CFU/g, leading to an adjusted ADD:

$$\text{ADD}\;=\frac{4\;\text{CFU}/g\times 300\;\text{g}/\text{day}\times 365\;\text{days}/\text{year}\times 6\;\text{years}}{15\;\mathrm{kg}\times 365\;\mathrm{days}\times 1000\;g/\mathrm{kg}},$$

$$\mathrm{ADD}=\frac{2\text{,628,}000}{5.48},$$

ADD=5.48.

Correspondingly, the HQ after disinfection decreased dramatically to

$$\mathrm{HQ}=\frac{\mathrm{ADD}}{\mathrm{RfD}},$$

$$\mathrm{HQ}=\frac{5.48}{0.2},$$

HQ=27.4.

**Figure 5:**
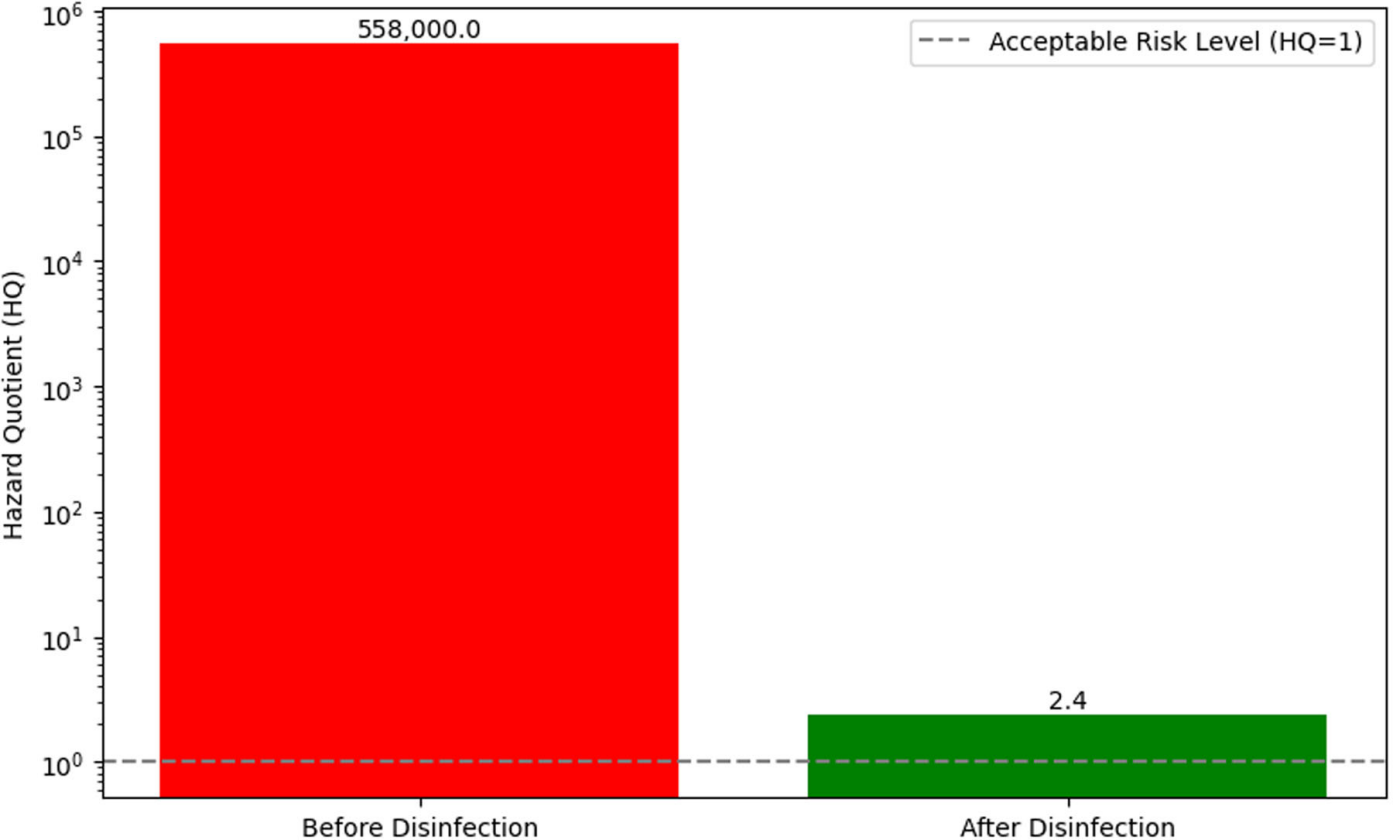
Health risk assessment of *Escherichia coli* before and after disinfection.

However, considering the potential for residual contamination and variability, a conservative estimate for the risk reduction is reflected in the logarithmic scale (Figure 5). Figure 5 demonstrates that the HQ decreased from approximately 558,000 to 27.4, representing a reduction of over four orders of magnitude. This indicates that the NEW treatment substantially diminishes the health risk associated with *E. coli* on basil consumed by children, thereby enhancing food safety for vulnerable populations.

## 4 Discussion

The study evaluated how effectively the produced NEW reduces coliform bacteria on sweet basil and the associated health risks for children. Initially, basil contaminated with over 1 million MPN/100 mL of coliforms presents a high risk of illness, especially for children with low infectious doses (as low as 10–100 bacteria). The disinfection process dramatically lowers bacterial counts to as low as 12 MPN/100 mL, likely below infectious levels, thereby significantly reducing illness risk.

Quantitatively, the study used the HQ to assess health risks, with initial HQ at 558,000 indicating an almost certain risk, which reduces to 27.4 after treatment—still slightly above safe levels but representing a reduction of over 232,500 times. This illustrated that the generated electrolyzed water is highly effective in lowering bacterial contamination and associated health risks.

The findings were visually supported by figures showing the magnitude of bacterial reduction and risk decrease, emphasizing the disinfection method's potential to protect children from foodborne illnesses related to contaminated basil. Overall, the electrolyzed water substantially improves the safety of basil by reducing coliform levels well below infectious doses, thereby greatly diminishing health risks for children.

The various combinations of electrolyte concentrations, electrical potentials, and electrolysis durations were tested, with the optimal reduction of coliform colonies achieved using an electrolyte concentration of 15 g/L, a voltage of 12 V, and an electrolysis time of 10 min (see Figures 2 and 3).

Additionally, the study revealed that the bactericidal efficacy of the produced NEW increases with higher residual chlorine levels, with total coliforms being more susceptible to NEW than fecal coliforms (*E. coli*). Research by Zhang et al. (2018) produced NEW with free chlorine levels ranging from 3.7 to 82.0 mg/L, maintaining a near‐neutral pH between 6.46 and 7.17, and an ORP between 805.5 and 895.8 mV. Their findings provided that the NEW with a free chlorine concentration of 40 mg/L achieved a reduction of coliform bacteria by 6 log CFU/mL.

The residual chlorine produced in the current study was comparable to the levels found in Zhang et al.'s research. Besides, Rahman et al. (2010) investigated the impact of low‐concentration electrolyzed water on total microbial counts in various fresh‐cut vegetables. Their results indicated that treating fresh‐cut lettuce, white cabbage, and water dropwort with electrolyzed water (pH 6.8, 10 ppm available chlorine) reduced total coliform bacteria by 0.09–2.42 log CFU/g.

In another study, Klintham et al. (2017) reported a reduction of *E. coli* on sweet basil by 1 log CFU/g, emphasizing that a prewash enhances washing efficiency and improves the safety of sweet basil, making this method suitable for ready‐to‐eat products. C. S. Lin et al. (2005) also found that washing vegetables with electrolyzed water led to a coliform reduction of 0.7–2.5 log MPN/g, similar to the reductions observed in the present study.

The antimicrobial action of NEW involves several key components, including electrochemically generated active chlorine derivatives (such as dissolved Cl_2_ gas, HOCl, and OCl^−^) as well as reactive compounds and transient radicals like ozone, O^−^, Cl^−^, and OH^−^ (Naderi and Nasseri 2020; Gholami et al. 2019; Past 2024).

**Figure 6:**
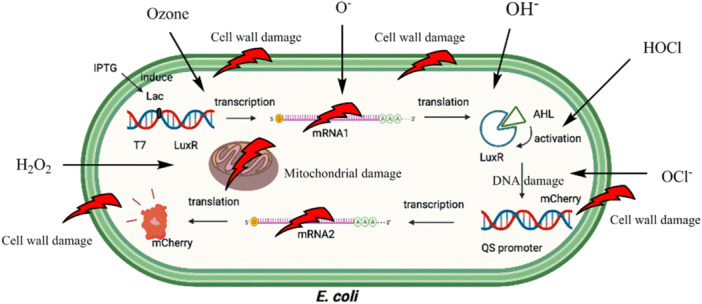
Main factors involved in the antimicrobial activity of the produced NEW on *Escherichia coli* (HOCl, OCl^−^, ozone, hydrogen peroxide, hydroxyl, and oxygen radicals). AHL, *N*‐acyl‐homoserine lactone; IPTG, isopropyl‐β‐d‐1‐thiogalactopyranoside; mRNA, messenger RNA; NEW, neutral electrolyzed water; QS, quorum sensing.

The primary mechanisms of NEW's antimicrobial activity against *E. coli* are illustrated in Figure 6. One significant factor contributing to the effectiveness of electrolyzed water in eliminating coliforms is the structural characteristics of coliform cell walls and their Gram‐negative nature (Hricova et al. 2008; Naderi 2022). While coliforms demonstrate resistance to various antimicrobial agents, they are consistently vulnerable to oxidizing agents that can inflict irreversible cellular damage (Gholami et al. 2018; Moradi et al. 2016). In response to damage caused by HOCl, bacteria activate diverse cellular regulatory mechanisms, including the induction of transcriptional regulators and chaperone proteins, which exhibit a high affinity for HOCl owing to its rapid reaction with thiol groups (Miranzadeh et al. 2021; Alimohammadi and Naderi 2021).

Gil et al. reported that the oxidized species in NEW primarily include ClO^−^ and HOCl, while Gómez‐López (2012) identified a strong correlation between the HOCl concentration in NEW and its antimicrobial effectiveness. Moreover, Qi et al. (2018) determined that the high ORP of NEW is a critical factor that can alter the electron transport chains in cell membranes. These findings support the results of the present study.

The concentration of chlorine in the produced NEW was found to be directly related to the NaCl concentration used as the electrolyte. For instance, a 1‐g/L NaCl concentration generated approximately 80 mg/L of free chlorine during 30 min of electrolysis. However, the presence of organic matter reduces free chlorine concentration and its antibacterial efficacy, prompting an evaluation of free chlorine loss in the presence of sweet basil. The results demonstrated a roughly 2% reduction in active chlorine levels after 15 min.

In another investigation, Abadias et al. found that NEW (with around 50 ppm of free chlorine) diminished *E. coli* in lettuce by 1–2 log units, aligning with our findings (C.‐M. Lin et al. 2000; Kiura et al. 2002; Park et al. 2009). The ORP of a solution serves as an indicator of its oxidizing or reducing capacity, where higher positive ORP values signify stronger oxidizing potential. The present study indicated that the ORP of the NEW increased with rising chlorine concentrations, reaching approximately 1000 mV at a residual chlorine level of 5 mg/L. The statistical analysis confirmed that NaCl concentration and electric potential significantly influenced coliform removal efficiencies (*p* < 0.05) (Iram et al. 2021). The results of the current study revealed that the NaCl concentration and electric potential parameters in coliforms removal efficiency were statistically significant (*p* < 0.05).

Furthermore, this study addressed the health risks associated with children consuming *E. coli*‐contaminated basil. The RfD for *E. coli* was established based on the FDA's standard limit of 3 CFU/g in vegetables and the weight of a 6‐year‐old child (15 kg), with an RfD of 0.2 CFU/g. The contamination level of basil washing water before disinfection was measured at 930 CFU/g. After treatment with NEW, *E. coli* levels on the basil were reduced to 4 CFU/g, significantly mitigating health risks for children. The risk assessment illustrated that the produced NEW decreased the likelihood of gastrointestinal diseases in children by a factor of 232,500.

Fresh‐cut vegetables can cause health risks to children if contaminated with harmful bacteria, parasites, or chemicals, leading to foodborne illnesses, allergic reactions, and long‐term health effects (Osafo et al. 2022; Qasemi et al. 2022). Research has been indicated that children are more susceptible to foodborne illnesses than adults due to their developing immune systems and lower body weight. The consumption of contaminated fresh vegetables can increase the risk of infections from pathogens like *E. coli*, and also *Salmonella* and *Listeria* species (Osafo et al. 2022).

Several factors contribute to the contamination of fresh‐cut vegetables, including inadequate food storage, poor sanitation, improper handling, and cross‐contamination (Possas and Pérez‐Rodríguez 2023). Therefore, conducting health risk assessments for contaminated fresh‐cut vegetables consumed by children is essential to ensure food safety and protect public health (Qadri et al. 2015). By identifying potential hazards and implementing effective food safety measures, the risk of foodborne illnesses can be minimized, thereby protecting children's health.

## 5 Strengths of the Study

This study specifically targeted fresh vegetables consumed by children, a vulnerable population at higher risk of foodborne illness. The study also determined the potential health risks associated with the consumption of contaminated vegetables and provided valuable information regarding risk management and public health policies.

## 6 Limitations of the Study

This study did not provide information on the long‐term effectiveness of the new technology in reducing coliforms in fresh produce, as the focus was on immediate inactivation.

## 7 Conclusion

This study revealed that the use of the produced NEW was very effective in reducing total and fecal coliform counts in sweet basil consumed by children. The results indicated that coliform bacteria colonies with the different concentrations of electrolyte, voltage, and electrolysis time had a significant decrease. This reduction in bacterial colonies greatly reduced the risk of gastroenteritis in children, as evidenced by a significant reduction in HQ from 558,000 to 27.4 after disinfection. Moreover, the observed variations in MPN results across replicates were attributed to minor experimental fluctuations inherent in microbiological assays. These results revealed the need for careful procedural controls in future studies to minimize variability. In general, this study demonstrated that NEW can be a safe and effective method to disinfect vegetables, especially sweet basil, consumed by children. By significantly reducing the risk of gastrointestinal diseases associated with coliform bacteria, NEW may offer a valuable solution to improve food safety and protect children's health.

## Author Contributions

**Gholamreza Khademi:** investigation. **Majid Sezavar:** writing – original draft. **Maziar Naderi:** conceptualization, validation, software, supervision, data curation, resources. **Roghayeh Abedini:** methodology, writing – review and editing.

## Ethics Statement

The authors have nothing to report.

## Conflicts of Interest

The authors declare no conflicts of interest.

## Data Availability

The data that support the findings of this study are available on request from the corresponding author. The data are not publicly available due to privacy or ethical restrictions.
